# Evaluating statistical learning methods for cell type classification and feature selection using RNA-seq data

**DOI:** 10.1186/1471-2105-15-S10-P26

**Published:** 2014-09-29

**Authors:** Hao Chen

**Affiliations:** 1 Department of Pharmacology, University of Tennessee Health Science Center, Memphis, TN 38106, USA

## Background

Single cell RNA-seq offers the opportunity to develop a systematic inventory of cell types in the brain.

## Materials and methods

We evaluated several statistical learning methods for classifying RNA-seq data using samples obtained from different brain regions as surrogates for single cell data. These include the ventral tegmental area (VTA, n=36), the nucleus accumbens core (AcbC, n=34) and shell (AcbS, n=30) [1]. We tested hierarchical clustering methods using different distance matrices (correlation, uncentered, abscor) and clustering algorithms. Multiscale bootstrap was used to evaluate the statistical significance of the clusters. We also tested the Bayesian Hierarchical Clustering method, which uses hypothesis testing to decide which cluster mergers increase the tree quality and calculates the optimum tree depth.

## Results

Both the Ward's algorithm and the Bayesian method correctly classified the majority of the samples. The reliability of the clusters were evaluated by repeated down sampling (without replacement). We found that sample sizes of 4-8/cluster were classified by the Ward's method with a very low error rate (0.02), while the error rate of BHC was slightly higher (0.11). We then compared Lasso and elastic net regularized logistic regression on the selection of genes that can be used as the molecular identity of each cluster. The Lasso method identified 15 genes (including Th, tyrosine hydroxylase) for the VTA cluster with cross validation error of 0.014. An elastic net regularized logistic regression model was also fitted for the VTA cluster. A two dimensional cross-validation procedure identified the parameters with the smallest cross validation error of 0.013. This model has 34 genes, among them are Th and Chrna4, two genes with high expression levels in the VTA. In addition, other models with slightly larger cross validation errors (~0.016) contain 35 -131 genes. We further fitted a multinomial elastic net model containing all three brain regions. The model with the smallest cross validation error (0.05) contained a set of 54 genes. Lastly, we tested the sparse hierarchical clustering method that combines clustering and feature selection.

## Conclusions

We found that although this method classified all VTA samples correctly, it failed to separate the AcbS and AcbC samples, potentially because the variable selection step removed some key information differentiating these two similar populations. In summary, these results provided guidance on the selection of statistical learning methods for studying single cell transcriptome data, where the cell type is unknown.
